# 

**Published:** 1864-12

**Authors:** 


### NOTICE.

Jackson, Jan. 1st, 1865.

*Dear Sir:*—The Annual Meeting of the Michigan Dental Association will be held in the City of Ypsilanti on Tuesday, January 24th, 1865, at 9 A. M.

The invitation is intended to be general, and it is hoped that the Profession will turn out and give their hearty support to the Association, which is so essential at the present time.

G. H. Mosher, *Secretary.*

### OHIO COLLEGE OF DENTAL SURGERY.

### SESSION OF 1864-'65.

The regular course of Lectures in this Institution, will commence on the first Monday of November, and close on the 20th of February.

---

### FACULTY.

JAS. TAYLOR, D. D. S. 171 Race Street.
Institutes of Dental Science.

J. TAFT, D. D. S. 172 Race Street.
Operative Dentistry.

GEO. EDWIN JONES, M. D. 481 West Seventh Street.
Anatomy and Physiology.

J. CHEESBROUGH,
Mechanical Dentistry.

H. A. SMITH, D. D. S. 118 West Sixth Street.
Chemistry and Metallurgy.

---

**Text Books.**—Gray's and Wilson's Anatomy; Carpenter's, Todd and Bowman's Physiology; Williams' Pathology; Fown's, Turner's, Graham's, or Brand and Taylor's Chemistry; Taft's Operative Dentistry; Richardson's Mechanical Dentistry; Harris' Principles and Practice Dental Science.

---

### TERMS OF ADMISSION.

Tickets must be procured at the beginning of the session.

| | |
|---|---|
| Tickets for the course, - - | $100 00 |
| Matriculation Fee, - - | 5 00 |
| Diploma Fee, - - - - | 30 00 |

---

### TERMS OF GRADUATION.

Two Courses, (including one of dissections,) with a dental pupilage.
Four years' reputable practice will be received as equal to one course.
An acceptabl thesis upon some subject on Dental science.
Must possess a good moral character, and be twenty-one years old.

For further information, address J. TAYLOR, *Dean.*
or J. TAFT, *Secretary.*

---

### ROBERTS' OS-ARTIFICIAL.

A substitute for Amalgam in filling badly decayed teeth; and used for reacting Pivot Teeth in badly decayed roots, also for filling over Sensitive Dentine to destroy sensibility, and as a non-conductor of heat, and for many other Dental Purposes.

For sale by all dealers in Dental Materials, and by the undersigned.

One-fourth ounce packages with directions sent by mail free of postage, on receipt of $1.

C. H. ROBERTS, M. D.,

[illegible], N. Y.

### PHILADELPHIA DENTAL COLLEGE

108 North Tenth St., above Arch.

SECOND ANNUAL SESSION, 1864-65.

---

### TRUSTEES.

PRESIDENT.

REV. RICHARD NEWTON, D.D.

SECRETARY.

R. SHELTON MACKENZIE, D.C.L.

PETER F. ROTHERMEL,

WILLIAM DULTY,

GEORGE J. ZIEGLER, M.D.,

GEORGE WILLIAMS,

ROBERT L. McCLELLAN, D.D.S.

J. L. SUESSEROTT, M.D., D.D.S..

COLSON HIESKELL,

S. FISHER CORLIES,

JAMES L. CLAGHORN,

EX-GOV. JAS. POLLOCK,

CHARLES S BECK, M.D.

HENRY CRUMLEY.

---

### FACULTY

C. A. KINGSBURY, D.D.S.,
Emeritus Professor of Dental Physiology and Operative Dentistry.

GEO. W. ELLIS, D.D.S.,
Professor of Dental Physiology and Operative Dentistry

THOS. WARDLE, D.D.S.,
Professor of Mechanical Dentistry and Metallurgy.

J. H. McQUILLEN, D D.S.,
Professor of Anatomy, Physiology and Hygiene.

J. FOSTER FLAGG, D.D.S.,
Professor of Institutes of Dentistry.

HENRY MORTON, A.M.,
Professor of Chemistry.

R. J. HOFFNER, D.D.S.,
Demonstrator of Operative Dentistry

WM. P. HENRY, D.D.S.,
Demonstrator of Mechanical Dentistry.

The Dispensary and Laboratory of the College will be open, and preliminary lectures will be delivered by one of the Professors every day during the latter half of October; commencing on the 17th instant, the lecture on Wednesday of each week, at 3 o'clock P.M., to be devoted to *Clinical* teaching. The regular Course of instruction will commence on the first Monday of November, and continue until the close of the ensuing February.

The lectures will be amply illustrated by the extensive and *valuable* collections of Anatomical, Pathological, and Mineralogical specimens, and the Philosophical and Chemical apparatus of the incumbents of the various Chairs, and every opportunity will be afforded in the Clinic and Laboratory for obtaining a *practical* knowledge of Operative and Mechanical Dentistry.

### FEES.

| | |
|---|---|
| Matriculation (paid but once) - - - - - - | $5 00 |
| Tickets for the Course, including the Demonstrators' - | 100 00 |
| Diploma - - - - - - - - - - - | 30 00 |

For further particulars, address J. H. McQUILLEN, Dean,
1112 *Arch Street*, *Philadelphia*.

July '64.

